# Soil Sample Assay Uncertainty and the Geographic Distribution of Contaminants: Error Impacts on Syracuse Trace Metal Soil Loading Analysis Results

**DOI:** 10.3390/ijerph18105164

**Published:** 2021-05-13

**Authors:** Daniel A. Griffith, Yongwan Chun

**Affiliations:** School of Economic, Political and Policy Sciences, The University of Texas at Dallas, 800 West Campbell Road, Richardson, TX 75080, USA

**Keywords:** geography of error, heavy metals, measurement error, resampling error, specification error

## Abstract

A research team collected 3609 useful soil samples across the city of Syracuse, NY; this data collection fieldwork occurred during the two consecutive summers (mid-May to mid-August) of 2003 and 2004. Each soil sample had fifteen heavy metals (As, Cr, Cu, Co, Fe, Hg, Mo, Mn, Ni, Pb, Rb, Se, Sr, Zn, and Zr), measured during its assaying; errors for these measurements are analyzed in this paper, with an objective of contributing to the geography of error literature. Geochemistry measurements are in milligrams of heavy metal per kilogram of soil, or ppm, together with accompanying analytical measurement errors. The purpose of this paper is to summarize and portray the geographic distribution of these selected heavy metals measurement errors across the city of Syracuse. Doing so both illustrates the value of the SAAR software’s uncertainty mapping module and uncovers heavy metal characteristics in the geographic distribution of Syracuse’s soil. In addition to uncertainty visualization portraying and indicating reliability information about heavy metal levels and their geographic patterns, SAAR also provides optimized map classifications of heavy metal levels based upon their uncertainty (utilizing the Sun-Wong separability criterion) as well as an optimality criterion that simultaneously accounts for heavy metal levels and their affiliated uncertainty. One major outcome is a summary and portrayal of the geographic distribution of As, Cr, Cu, Co, Fe, Hg, Mo, Mn, Ni, Pb, Rb, Se, Sr, Zn, and Zr measurement error across the city of Syracuse.

## 1. Introduction

The geography of error (arising from, e.g., calculation, sampling, measurement, specification, and stochastic sources) dates back many decades, with Goodchild and Gopal’s [1] book, followed by the first International Symposia on Spatial Accuracy Assessment in Natural Resources and Environmental Sciences convened in Williamsburg, VA, in 1994 (http://www.spatial-accuracy.org/History, accessed on 8 February 2021), signifying more concerted geospatial research efforts to address this theme. The history of this topic parallels that of popular applied statistics in general, which evolved from reporting only central tendency values to also reporting their associated uncertainties (e.g., margins of error), which became a common practice only after 1928. In parallel, maps began including error statements, mostly after 1941, although they were global map-wide ones. However, reporting a global error measure on an increasing number of maps furnishes little knowledge and understanding about the geography of error in general (even though a meta-analysis might provide some insights). Meanwhile, as geostatistical analysis became more prevalent (the software packages GEO-EAS [2] and GSLIB [3] appeared at the beginning of the 1990s), prediction error maps began accompanying krigged surface (i.e., mean response) maps, a practice that continues today. The principal disclosure of this geography of error exception is that its map pattern relates to the underlying sampling network, which almost always is visible in a prediction error map. Hexagonal-tessellation stratified random spatial sampling (e.g., [4]) error constitutes another exception, with a primary concern of its geography being the relationship between spatial landscape coverage and sampling error. Current georeferenced data releases, such as those from the United States (USA) American Community Survey (ACS), include sampling errors, furnishing an increasing number of databases to study the geography of sampling error in a more comprehensive way.

The objective of this paper is to contribute to this geography of error literature, focusing on combinations of measurement (i.e., soil geochemistry content analysis accuracy/precision), sampling (i.e., the selection of only a tiny fraction of soil in a geographic landscape), and specification (i.e., the correctness of assumptions and/or functional forms of equations for an analysis) errors in the context of spatial autocorrelation, a fundamental feature of geospatial data. Its empirical analyses and simulation experiments exploit a unique database containing analytical measurement errors (i.e., uncertainty introduced by chemical assay procedures) for trace metals contaminating soil in the city of Syracuse, NY, USA.

## 2. Background

Errors in georeferenced data can occur in both of their components: location and attribute. The geography and GIScience literature contains extensive investigations about location errors in various contexts, including geocoding (e.g., [5,6]) and raster modeling (e.g., [7]). This literature also includes studies about georeferenced data attribute errors. Griffith et al. [8] discuss four major sources of georeferenced data errors, namely sampling error, measurement error, specification error, and analytical assaying error. The literature addresses these first three sources for attribute errors. For example, Griffith et al. [9] argue that sampling error of estimates is heterogeneous across spatial units, specifically census tracts, and tends to correlate with both the size of tracts and their socio-economic characteristics. Wang et al. [10] discuss sampling as a major source of error, and present a sampling approach to reduce error variances. In addition, Leung et al. [11] discuss a general error analysis framework concerning georeferenced data measurements, whereas many literature entries (e.g., [12,13,14]) recognize model specification error. In contrast, assaying errors have not been a popular topic in spatial analysis and/or spatial statistics, although they generally occur in field surveys of, for example, soil [15,16]. Kriging is one spatial analysis topic that does not avoid referencing assaying errors; it links these errors to the nugget effect [17]. Otherwise, assaying errors are rarely investigated in spatial analysis.

Griffith et al. [8] discuss ways to enhance research about uncertainty. They focus on the following four research themes: visualizing error; spatial patterns and spatial modelling acknowledging error; spatial data aggregation; and data quality. Visualizing error, the first theme, can improve the current understanding about spatial patterns of information uncertainty. Research dealing with mapping uncertainty presents various approaches focusing on mapping observations together with their data quality information [18,19]. Recently, Koo et al. [20] discussed a framework for uncertainty mapping. Nevertheless, the common protocol has two side-by-side maps, displaying a geographic distribution of values juxtaposed with a geographic distribution of individual value uncertainties, because concurrently exhibiting the two sets of information in a single map tends to be overwhelmed by the portrayal of a large amount of data in a limited two-dimensional space.

Spatial patterns and spatial data modelling constitute a second research theme that can benefit from incorporating uncertainty into spatial analysis. Sun et al. [21] discuss a map classification technique that seeks to avoid adverse impacts by the presence of uncertainty, one of which is the possibility of a different map pattern outcome. They present a new map classification approach incorporating uncertainty, demonstrating it with American Community Survey data, which is accompanied by sampling error measures. Meanwhile, Koo et al. [22] and Mu and Tong [23] present alternative approaches to incorporating uncertainty into map classifications. New approaches to spatial modelling recognizing uncertainty, going beyond widely recognized model specification issues such as omitted variables [24], also need development. Hu et al. [14] furnish one promising effort that models the existence of a mixture of positive and negative spatial autocorrelation in spatial data, showing how successfully accounting for such a mixture pattern can enhance a spatial analysis.

A third research theme, spatial data aggregation, can also raise uncertainty issues. The modifiable areal unit problem (MAUP) describes a convolution of spatial analysis, with observations based on polygon area units [25]. Lee et al. [26] present simulation experimental results demonstrating how spatial autocorrelation levels may be affected by areal unit aggregation (both geographic resolution and zonation). Despite these spatial data aggregation issues being recognized in the literature for decades now, a universally acceptable solution to the MAUP remains elusive for geographic analysis. Finally, Griffith et al. [8] discuss the importance of spatial metadata for data quality so that users understand the accuracy level of data and, when necessary, more cautiously assess spatial analysis results.

Given this preceding context, this paper investigates assaying measurement errors of heavy metal amounts in soil sample observations. It focuses mainly on the geographic patterns that materialize from mapping individual soil samples with point locations and then further aggregates these data by census tracts. In addition, this paper explores both individual and a mixture of different error types with simulation experiments. By doing so, its principal knowledge contribution is to the geography of error literature.

## 3. About the Data

**Figure 1:**
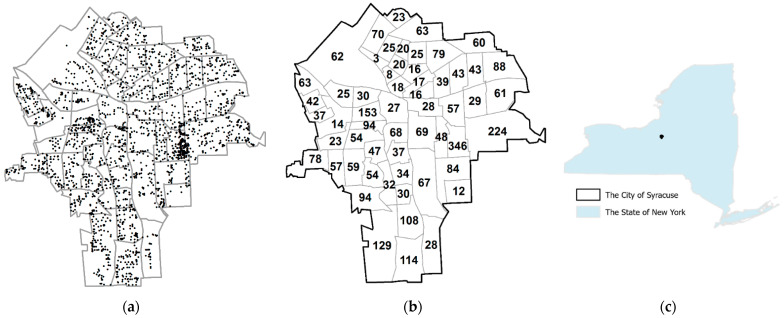
Syracuse, NY. (**a**) The geographic distribution of trace metal soil samples overlaid with a polygon map of the 2000 United States census tract boundaries. (**b**) The distribution of number of soil samples with analytical assay error measurements. (**c**) The study area in the state of New York.

A total of 3628 useful surface soil samples were collected across the city of Syracuse, New York, USA, mostly during the summers of 2003 and 2004, under the auspices of the National Science Foundation (BCS-0552588), with Institutional Review Board (IRB) oversight by that organization for Syracuse University, then the University of Miami, and finally the University of Texas at Dallas. Of these, 3324 unique location samples―multiple samples for the same locations were averaged by location―had useful (i.e., positive) analytical assay error values (Figure 1); two of these soil samples had locations outside of Syracuse, resulting in their being removed from census tract resolution spatial analyses. This dataset contained a rich set of observations, with extensive sample coverage across the city of Syracuse, including measurements for 15 heavy metals and corresponding assay errors with accurate point location tags. Accordingly, it furnished a rare opportunity to investigate geographic patterns of assay error at the individual point level as well as to compare these individual results with ones at an aggregated geographic resolution, specifically the census tract level. Currently, this type of dataset is scarce.

A NITON XL-700-series x-ray fluorescence (XRF) instrument (NITON Corporation 900 Middlesex Turnpike, Billerica, MA 01821)was used in a chemistry laboratory to measure 15 trace metals (As, Cr, Co, Cu, Fe, Hg, Mn, Mo, Ni, Pb, Rb, Se, Sr, Zn, and Zr; see Table 1) in these soil samples, based on 120 s testing time and NIST 2711 standard reference materials (SRM). Assaying also supplied standard deviations for each of these quantities (i.e., analytical assay errors) by soil sample. Calculated quantities are in milligrams of trace metal per kilogram of soil, or parts per million (ppm). Table 1 summarizes detection and natural background thresholds for these metals as well as the number of useful samples (i.e., with at least a non-negative assay trace metal measurement quantity) exceeding either a published maximum permissible level (MPL; [27]) or the worldwide average contamination/risk threshold [28]; all soil samples have an analytical error calculation, but not all have a valid (i.e., non-negative) trace metal quantity calculation. A number of already-published studies ([29,30,31,32,33,34]) summarize targeted analyses of the geographic distribution of this sample of trace metal measurement quantities and/or their location error. In contrast, assaying-furnished error standard deviations for all soil samples constitute a dataset yet to be analyzed, until now.

A useful expectation is that a log-normal distribution describes the ordered distribution of soil contaminant errors (after [39]; also see [40]), especially for a cross-sectional study. Diagnostic results appearing in Table 2 imply that a log-normal approximation furnishes a suitable, albeit not perfect, description of the sample trace metal errors studied here. With so little residual variance (Table 2), any residual soil sample geographic resolution level spatial autocorrelation that is present has little impact upon variance estimates; this topic constitutes an appealing future research theme. The classical form of the log-normal random variable implies the following theoretical standard deviation (std), acknowledging that its minimum is zero (i.e., variance is non-negative):

$$\mathrm{LN}\left(y_{o}+\hat{\delta }\right)={\hat{\mu }}_{o}=\hat{\alpha }+\hat{\beta }\left\{\Phi^{-1}\left[\frac{r_{o}-3/8}{n+1/4}\right]\right\}+{\hat{\varepsilon }}_{o},\;\mathrm{if}\;y_{o}\;\mathrm{is}\;\mathrm{from}\;\mathrm{the}\;\mathrm{sample}\;\mathrm{being}\;\mathrm{studied},\;$$

$$\mathrm{LN}\left(y_{o}+\hat{\delta }\right)={\hat{\mu }}_{o}=\hat{\alpha }+\hat{\beta }\left\{\Phi^{-1}\left[\frac{r_{o}-3/8}{n+1+1/4}\right]\right\}+{\hat{\varepsilon }}_{o},\;\mathrm{if}\;y_{o}\;\mathrm{is}\;\mathrm{from}\;\mathrm{outside}\;\mathrm{of}\;\mathrm{the}\;\mathrm{sample}\;\mathrm{being}\;\mathrm{studied},\;$$

$${\hat{\sigma }}_{\mathrm{LN}\left(y_{o}+\hat{\delta }\right)}^{2}={\hat{\sigma }}_{\varepsilon }^{2}=\;\mathrm{MSE},\;$$

$${\hat{y}}_{o}=-\hat{\delta }+{\;e}^{{\hat{\mu }}_{o}+{\hat{\sigma }}_{\mathrm{LN}\left(y_{o}+\hat{\delta }\right)}^{2}/2},\;\mathrm{and}\;$$

$${\hat{\sigma }}_{y_{o}}=\sqrt{\left[e^{2{\hat{\mu }}_{o}+{\hat{\sigma }}_{\mathrm{LN}\left(y_{o}+\hat{\delta }\right)}^{2}}\right]\left[e^{{\hat{\sigma }}_{\mathrm{LN}\left(y_{o}+\hat{\delta }\right)}^{2}}-1\right]},\;$$

where r_o_ is the rank of the existing/new value, y_o_, MSE denotes the appropriate regression mean squared error (see Appendix B), and Φ denotes the standard normal random variable’s cumulative distribution function (the mean and variance of these n or n + 1 z-scores respectively are 0 and 1). This data feature suggests that theoretical standard errors may be posited for each of the 15 trace metal variances studied in this paper (based on Appendix B, Table A1). These quantities support a resampling-based uncertainty measure for each of the quantified analytical assay standard deviations.

Table 3 summarizes selected descriptive spatial statistics about the trace metal analytical errors, recognizing that individual soil contaminants, because of their positive skewness (e.g., the minimum is zero), often are approximately log-normally distributed (e.g., [40]). In general, their latent spatial autocorrelation is positive and weak, which is sensible given that analytical error basically should be locationally independent across samples. Similarities common to nearby sample soil compositions could introduce some spatial autocorrelation; however, the assaying sequence should not.

## 4. Dimensions of Analytical Assay Error

One relevant research question asks whether or not the geographic distribution of trace metal soil sample analytical assay measurement error has distinct dimensions. These errors for the 15 heavy metals were analyzed with factor analysis. Table 4 reports factor loadings of the four prominent uncovered data dimensions (after being subject to a varimax rotation), which summarizes a simple structure (i.e., varimax rotated) version for them. Both the point and census tract averaged data render the same four dimensions, which account for more than 90% of the total generalized variance for the 15 trace metal analytical assay errors, with all of these errors positively correlated with their respective data dimensions. In accordance with their toxicity to living organisms, a subset of these heavy metals can be ordered as follows: Hg > Cu > Zn > Ni > Pb > Cr > Fe > Mn [41]. These four dimensions are as follows: (1) Co, Cu, Hg, Ni, Se, and Sr; (2) Co, Fe, Mn, and Rb; (3) As, Pb, and Zn; and, (4) Mo and Zr. This first dimension most likely embodies the most toxicity. Factor-1 essentially reflects variability patterns arising from biosolids and manure usage coupled with airborne vehicle pollution. Factor-2 signals variability patterns arising from the use of modern yard landscape organic and mineral fertilizers. Factor-3 signifies variability patterns typical of urban soil contamination arising from the use of outdoor wood and metal material treatments coupled with pesticide chemicals for landscape plant protection. Factor-4 may mirror variability patterns attributable to a geogenic dimension, given that lower amounts of Zr tend to be found in glacial drift soils (e.g., drumlins), whereas Mo contamination of soils is widespread in historically industrial places, particularly those that produced steel alloys (the 100+ years of production continues in Syracuse to this day in the form of specialty steel) (See Appendix C).

**Figure 2:**
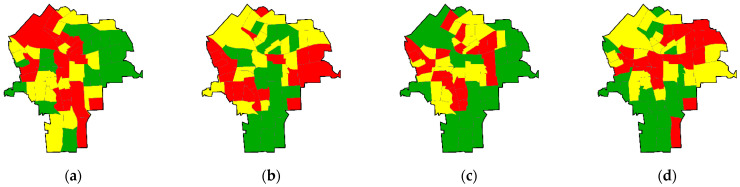
Tertile choropleth maps for the four aggregated trace metal error dimensions across Syracuse, NY; red denotes relatively high factor scores, yellow denotes intermediate factor scores, and green denotes relatively low factor scores. (**a**) Factor-1 (MC = 0.185, GR = 1.038); (**b**) Factor-2 (MC = 0.242, GR = 0.800); (**c**) Factor-3 (MC = 0.162, GR = 0.725); (**d**) Factor-4 (MC = 0.300, GR = 0.618).

Figure 2 portrays the geographic distributions of the four trace metal error dimensions. The red census tracts in Figure 2a reflect the interstate expressways transecting the city. The red census tracts in Figure 2b focus on the locations of the pre-city settlements of the village of Syracuse and the town of Salina during the early 1800s. The green census tracts in Figure 2c highlight much of the low density housing and other low population density land use sections of the city. Finally, the red census tracts in Figure 2d basically align with major drumlins across the city. Overall, these four map patterns are consistent with the preceding trace metal dimension interpretations. Furthermore, the spatial autocorrelation exhibited by these four dimensions signals the swarthiness tendencies rather than any coherent geographic clusters depicted by their map patterns.

**Figure 3:**
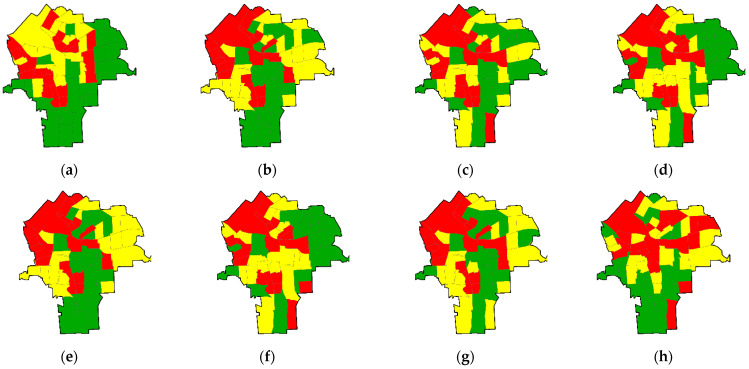

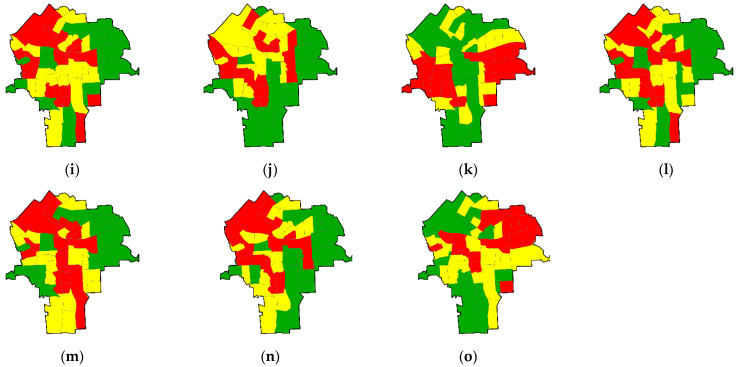
Tertile choropleth maps portraying the 15 trace metal analytic measurement error geographic distributions (census tract resolution): (**a**) As, (**b**) Cr, (**c**) Co, (**d**) Cu, (**e**) Fe, (**f**) Pb, (**g**) Mn, (**h**) Hg, (**i**) Mo, (**j**) Ni, (**k**) Rb, (**l**) Se, (**m**) Sr, (**n**) Zn, and (**o**) Zr; red denotes relatively high errors, yellow denotes moderate errors, and green denotes relatively low errors.

Figure 3 presents the geographic distributions of the analytic assay measurement errors for the 15 trace metals using a quantile classification scheme. These maps generally confirm the results of the dimension analysis. The maps for the metals in Factor-1 (i.e., Co, Cu, Hg, Ni, Se, and Sr) have a similar pattern, with high values in the northwest and southeast areas and low values in the northeast area. The four metals in Factor-2 (i.e., Co, Fe, Mn, and Rb) have a similar pattern, with the first three having high values in the northwest area and low values in the south area; Rb has a slightly different geographic pattern from the others in Factor-2, with high value clusters in the east as well as the west side and low values through the central north-south axis of the city. The three metals in Factor-3 (i.e., As, Pb, and Zn) have a similar geographic pattern. In particular, the maps for As and Ni are almost identical with the quantile taxonomy, depicting high values occupying the center and the northwest areas and low values occurring in the northwest and south areas. The maps for Mo and Zr generally show high values in the northern part and low values in the southern part of the city.

## 5. Mapping Analytical Assay Measurement Error and Sampling Error

Although the choropleth maps present the spatial patterns of the assay errors for the heavy metals, they do not consider the uncertainty of these assay errors. Studies discuss that a map classification result may not be robust when the uncertainty of observations is not considered [42,43]. This section presents choropleth maps for the heavy metals utilizing the approach presented in Koo et al. [22] and the separability index [21], which consider observed values and their uncertainties simultaneously in map classification. The choropleth mapping methods are implemented in the SAAR software package that utilizes R functions through R.NET [44]. It is available from https://thesaar.github.io/ (accessed on 30 April 2021).

**Figure 4:**
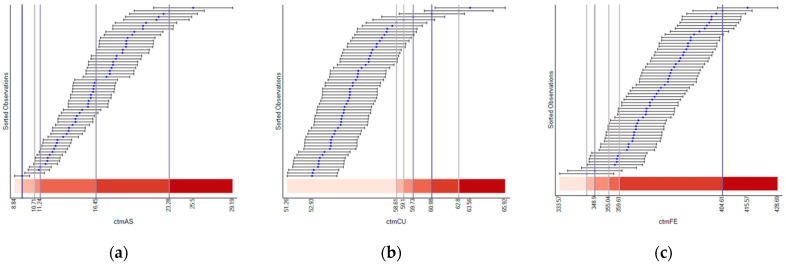
Illustrative heavy metals classification results for assay error and their corresponding theoretical analytical measurement error: (**a**) As, (**b**) Cu, and (**c**) Fe.

**Figure A2:**
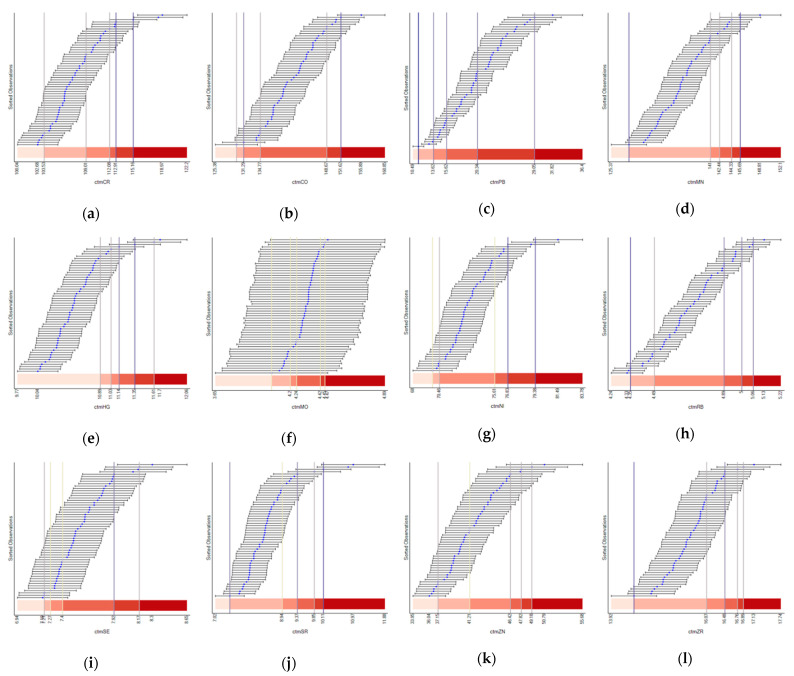
Classification results of assay errors and the corresponding theoretical analytical measurement error for three selected heavy metals: (**a**) Cr, (**b**) Co, (**c**) Pb, (**d**) Mn, (**e**) Hg, (**f**) Mo, (**g**) Ni, (**h**) Rb, (**i**) Se, (**j**) Sr, (**k**) Zn, and (**l**) Zr.

Figure 4 presents visual representations of classification results for the assay errors and their theoretical analytical measurement errors for three selected heavy metals (As, Cu, and Fe). These three heavy metals are highly associated with Factors 3, 1, and 2, respectively, reported in the dimensional analysis section (Table 4). Note that plots for the other heavy metals are presented in Appendix D (Figure A2). In these plots, blue dots represent assay errors for the census tracts, and the corresponding bars represent the 95% confidence intervals calculated with analytical measurement errors. The analytical measurement error is relatively consistent across the census tracts. This consistent pattern is conspicuous for Cu and Fe. In contrast, the analytical measurement error for As tends to increase as assay error increases. In other words, the length of the bars gets longer as the As value increases. Nevertheless, the analytical errors for two consecutive observations in the graph are similar. Because of this error consistency, at least between consecutive observations, the assay error values (blue dots) profoundly dominate, whereas the analytical error has little or no impact on the classification results. The classification break points (the vertical bars) appear where the assay error value separations are sizeable. The plots for the other heavy metals have this same pattern.

**Figure 5:**
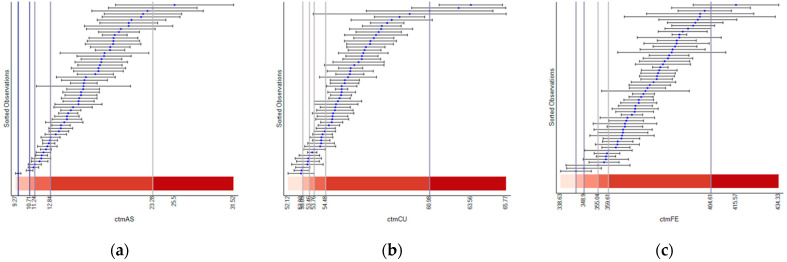
Illustrative heavy metals classification results for assay error and their corresponding resampling error: (**a**) As, (**b**) Cu, and (**c**) Fe.

**Figure A3:**
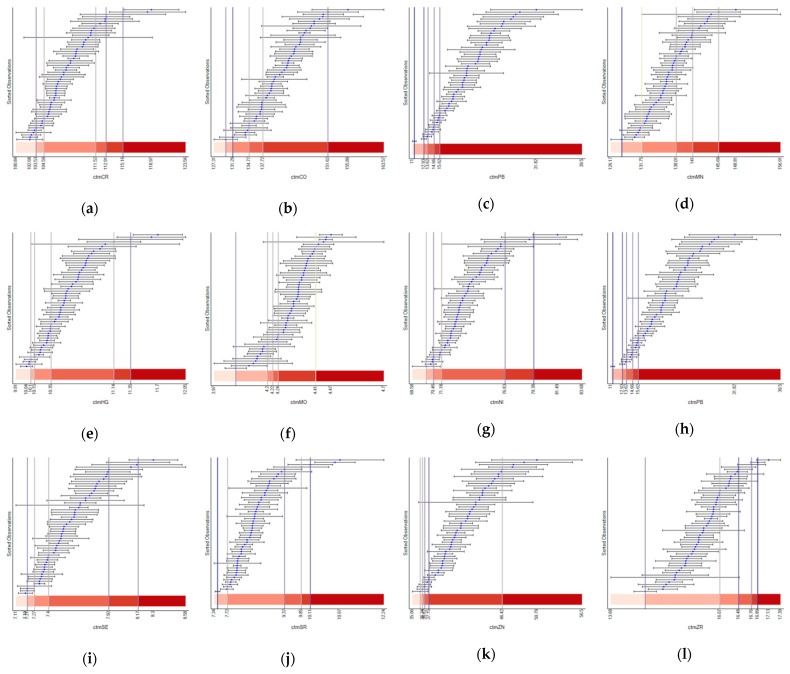
Classification results of assay errors and the corresponding resampling error for three selected heavy metals: (**a**) Cr, (**b**) Co, (**c**) Pb, (**d**) Mn, (**e**) Hg, (**f**) Mo, (**g**) Ni, (**h**) Rb, (**i**) Se, (**j**) Sr, (**k**) Zn, and (**l**) Zr.

Figure 5 presents the classification results based upon the assay error and resampling error. The resampling error varies across census tracts more than the analytical error. For example, the resampling error of Cu varies, while its analytical error is relatively constant. All heavy metals have a similarly larger variation in their resampling errors (Figure 5 and Figure A3). This resampling error variation has an impact on classification results. For Cu, although most observations fall into the first class in Figure 4b, most observations fall into the fifth class in Figure 5b. For As, most observations fall into the fifth class in Figure 5a, whereas observations are relatively equal in number across the fourth and fifth classes in Figure 4a.

**Figure 6:**
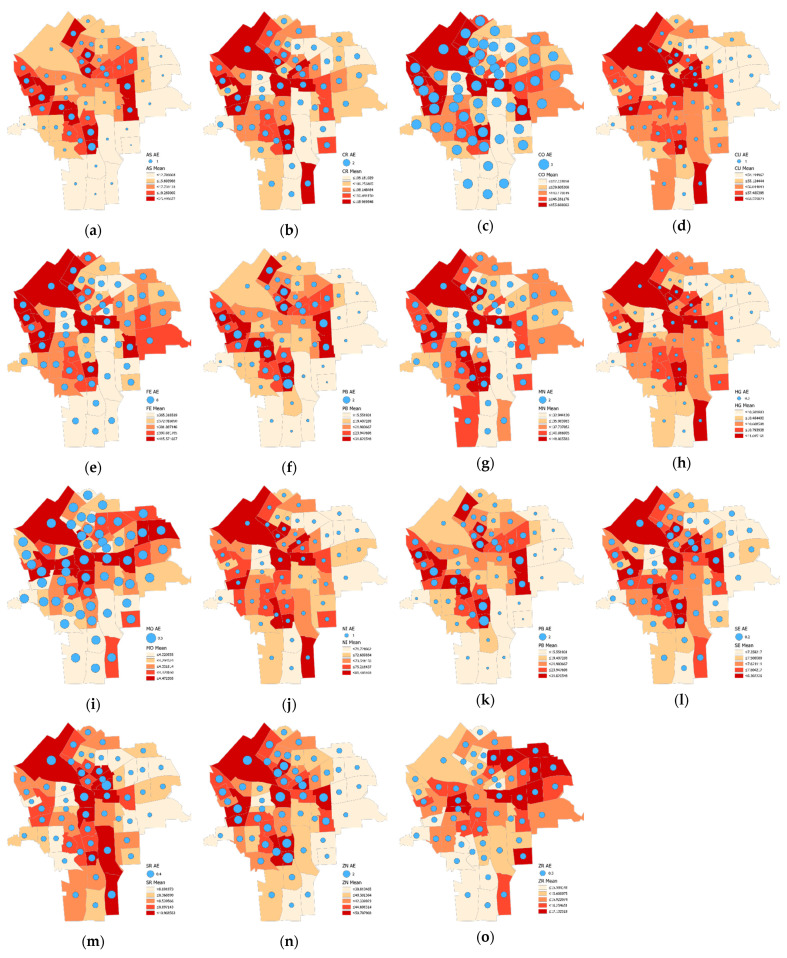
Choropleth maps with superimposed theoretical analytical measurement error magnitude proportional circles portraying each of the 15 trace metals (census tract resolution): (**a**) As, (**b**) Cr, (**c**) Co, (**d**) Cu, (**e**) Fe, (**f**) Pb, (**g**) Mn, (**h**) Hg, (**i**) Mo, (**j**) Ni, (**k**) Rb, (**l**) Se, (**m**) Sr, (**n**) Zn, and (**o**) Zr.

Figure 6 presents the geographic distribution of the census tract aggregated simulated log-normal analytical measurement error for each metal. The magnitudes of the errors are represented with proportional circle sizes (the symbol sizes in the legends for their corresponding labels), whereas the choropleth maps represent mean values for the heavy metals. Some metals, such as Co (Figure 6c), display relatively sizeable error dispersions, whereas others, such as Hg (Figure 6h), display relatively small error dispersions, and yet others, such as As (Figure 6a), display more of a mixture of dispersion magnitudes. Regardless, all are well described by a log-normal distribution (Table 2).

**Figure 7:**
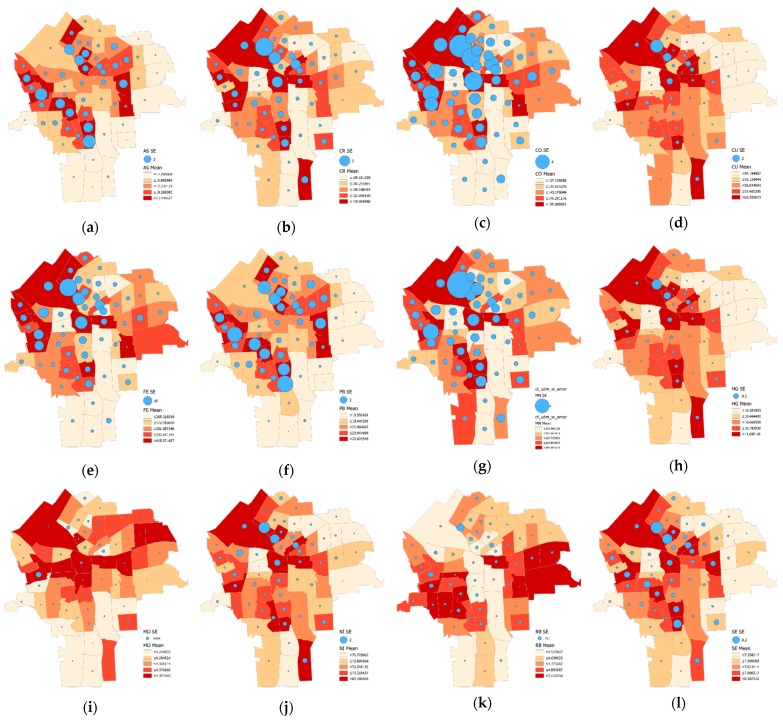

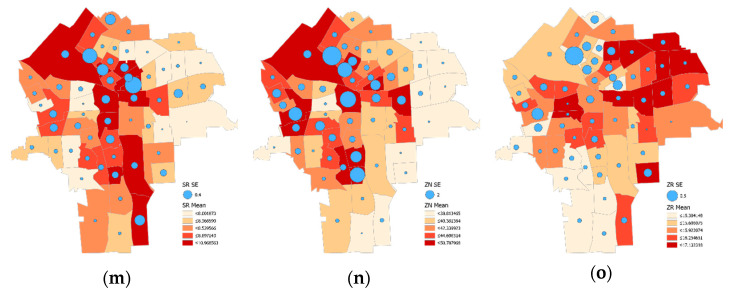
Choropleth maps with superimposed resampling error magnitude proportional circles portraying each of the 15 trace metals (census tract resolution): (**a**) As, (**b**) Cr, (**c**) Co, (**d**) Cu, (**e**) Fe, (**f**) Pb, (**g**) Mn, (**h**) Hg, (**i**) Mo, (**j**) Ni, (**k**) Rb, (**l**) Se, (**m**) Sr, (**n**) Zn, and (**o**) Zr.

Figure 7 reveals that the resampling error dispersion tends to be substantially larger than its corresponding analytical measurement error. Sampling error across the Figure 7 maps partially reflects variation in the post-tabulated sample sizes, which is far from constant. Accordingly, many maps (e.g., Figure 7c,h, respectively portraying Co and Hg) have relatively wide ranges of dispersions, which are quite conspicuous. Some (e.g., Figure 7b,k) have relatively narrow ranges of small dispersions, whereas none have relatively constant and more substantial resampling errors (similar to Figure 6b).

The geographic error variability displays some noticeable features and evokes several implications. First, analytic measurement error generally is stable across census tracts. Most of the heavy metals have essentially the same analytical error for all of the census tracts, although some heavy metals (As, Pb, and Zn) show an increasing pattern that covaries with the assay error. Second, the resampling error varies considerably, unlike the analytical measurement error. The geographic distributions of the resampling error show pattern similarity to the factors uncovered as data analytic dimensions of the assay error. The heavy metals that are mainly associated with the same factor in Table 4 have a map pattern similar to that of the resampling error. For example, the geographic patterns for Co, Fe, Mn, and Hg, which mainly contribute to Factor-2, are alike. This outcome may imply that resampling error results can be affected by samples in an areal unit, consequently affecting its geographic pattern.

## 6. Error Mixtures in Data: Some Simulation Experiments

This section summarizes output from exploratory simulation experiments addressing the sensitivity of map patterns to analytical assay measurement error (via sampling from appropriate log-normal random variables), sampling error (via bootstrap resampling), and their combination. Table 4 reports census tract resolution summary results for the observed data. Concluding comments focus on specification error with regard to estimating the nature and degree of spatial autocorrelation in geographic dimensions of data.

### 6.1. Geographic Dimension Sensitivity to Analytical Assay Measurement Error

Generation of the assaying sensitivity analysis simulation experiment results summarized in Table 5 utilized random samples from appropriate heavy metal log-normal distributions for each of the 3322 individual soil samples located in the city of Syracuse. These census tract averages then were georeferenced data input to a varimax-rotated factor analysis. Results for the data analytic factor structure of the 15 Syracuse, NY, trace metal measurement errors imply that changes in assay measurement error based on theoretical frequency distributions corrupt findings in subtle ways (e.g., average Factor-2 and Factor-3 loadings decrease, but not significantly), although it preserves simple structure as well as the dimensional clustering of heavy metals. Nevertheless, the percentage of variance accounted for by each dimension remains essentially the same as that for the observed data.

### 6.2. Geographic Dimension Sensitivity to Sampling Error

This resampling experiment addressing sensitivity to soil sampling error employed a bootstrap census tract tessellation stratified random sampling (with replacement) design. Table 6 summarizes sampling error resampling findings for the data analytic factor structure of the 15 Syracuse trace metal measurement errors. Sampling error corrupts results in various ways. Four heavy metals exhibit a propensity to load onto two factors, compromising simple structure. Now Zn loads onto the first dimension (which arguably occurs for the collected data), and Rb fails to load onto any of the first four dimensions. Prominent loadings for the second and third dimensions markedly decrease (although not significantly). Meanwhile, as with the assaying simulation findings, the percentage of variance accounted for by each dimension remains essentially the same as that for the observed data.

**Figure 8:**
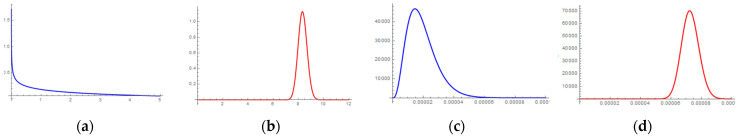
Specimen gamma (left) and beta (right) distributions. (**a**) α = 0.065, 1/β = 62; (**b**) α = 0.489, 1/β = 1337; (**c**) α = 4.11 β = 212,688; (**d**) α = 163.19, β = 2,239,631.

A third experiment, addressing sensitivity to specification error, employed the gamma distribution (with $\mathrm{LN}\left[\frac{r_{o}-3/8}{n-r_{o}+5/8}+0.25\right]$ as its covariate; representing minimal specification error) because it furnishes a competitive empirical distribution to the log-normal one. It also employed the beta distribution (with $\mathrm{LN}\left[\frac{r_{o}-3/8}{n-r_{o}+5/8}\right]$ as its covariate; representing noticeable specification error), which provides a conceptually appealing alternative, given that the trace metal quantities are proportions in ppm. It additionally employed the uniform distribution (with p_i_ = $\frac{r_{i}-3/8}{n+1/4}$ as its covariate; representing severe specification error), whose probabilities are much smaller for the concentration portion and much larger for the right-hand tail of the positively skewed empirical frequency distribution. Based upon order statistics combined with six-sigma theory—e.g., covariate values have a variable transformed beta distribution with variance $\sqrt{\frac{r_{i}\left(n-r_{i}+1\right)}{\left(n+2\right){\left(n+1/4\right)}^{2}}}$ for the *i*th rank—sampling draws were from the uniform distribution over the interval

$$\left[p_{i}\;–\;3\sqrt{\left\{p_{i}\left(1-p_{i}\right)+\frac{33}{64{\left(n+\frac{1}{4}\right)}^{2}}\right\}/\left(n+2\right)]},\;p_{i}+3\sqrt{\left\{p_{i}\left(1-p_{i}\right)+\frac{33}{64{\left(n+\frac{1}{4}\right)}^{2}}\right\}/\left(n+2\right)]}\right],$$

where $p_{i}$ (which also is the covariate) denotes the empirical cumulative distribution function probability for soil sample i. In each case, 10,000 analytical assay measurement error samples were drawn. Table 7, Table 8 and Table 9 summarize averaged varimax-rotated factor analysis results from these experiments. Meanwhile, Figure 8 portrays selected extreme gamma and beta distributions embraced by these simulations.

Approximating the analytical assay measurement error that is a proportion restricted to the interval [0, 1] with a log-normal distribution whose support is the interval [0,∞) appears to introduce little specification error, most likely because the skewness of heavy metal error tends to concentrate its values very close to zero. However, specification error introduced by replacing this log-normal with its gamma distribution competitor [45] corrupts a number of results (see Table 7): Mn loads onto the first rather than the second dimension; Pb and Zn fail to load onto any of the first four dimensions; all of the prominent loadings are lower, some substantially so, verging on statistical significance; and the percentage of variance accounted for decreases noticeably for the first three dimensions. Figure 8 portrays the scatterplot association between the log-normal and gamma individual heavy metal goodness-of-fit regression (pseudo-)R^2^ values (also see Appendix E, Table A3); overall, the gamma assumption appears to be inferior to the log-normal assumption.

**Figure 9:**
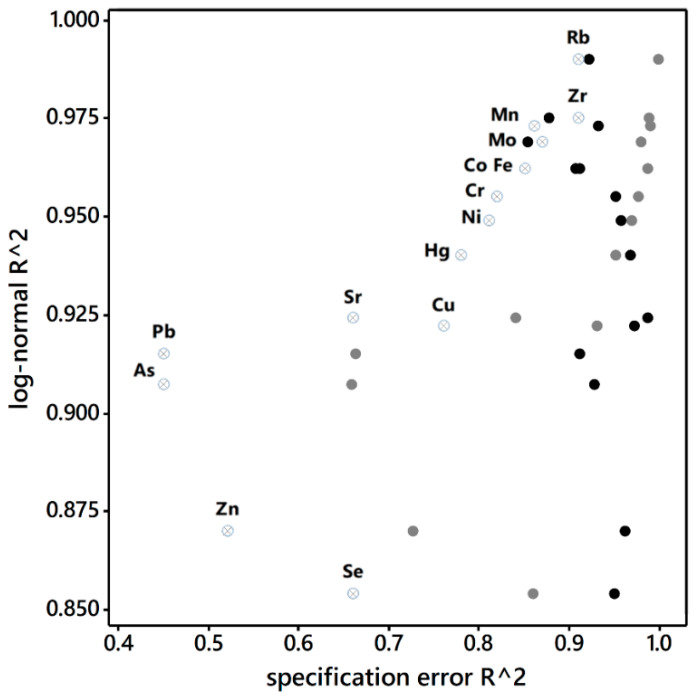
Goodness-of-fit (pseudo-)R^2^ scatterplots labelled by heavy metal (each label pertains to a horizontal row of three scatterplot points). Denoted distribution results are: solid black circles denote gamma; solid gray circles denote beta; and, Hebrew pictograms tet (⨂) denote uniform.

Meanwhile, although the beta distribution and analytical assay measurement error share the same support interval, the beta distribution’s performance is poorer than that produced by the gamma assumption (Table 5, Table 7 and Table 8). Here the first dimension gains Co and Mn and loses Hg and Sr. The second dimension resembles the original third dimension, whereas the third dimension lacks substance. Zn fails to load onto the fourth dimension; more specifically, Fe, Hg, Rb, Se, and Zr fail to load onto any of the first four dimensions. Again, all of the prominent loadings are lower, some substantially so, verging on statistical significance. Figure 9 also portrays the scatterplot association between the log-normal and beta individual heavy metal goodness-of-fit regression (pseudo-)R^2^ values (see Appendix E, Table A3); overall the beta appears inferior to the gamma, and markedly inferior to the log-normal, assumption.

Figure 9 endorses the contention that the uniform distribution represents the most extreme specification error assumption studied here. Table 9 reveals that the simulation error is negligible because selection is from relatively narrow intervals (regardless of the use of the six-sigma principle). This assumption essentially preserves the multivariate statistical structure of Table 5 but with an overfitting cost (i.e., analyses over-emphasize assumption-specific information). The second and third dimensions switch (which is not surprising, given that the respective percentages of accounted variance are almost the same), and many of the loadings decrease somewhat. Nevertheless, a number of the bivariate regression R^2^ values are only moderate in degree and hence relatively low (see Appendix E, Table A4), with corresponding coefficients that deviate considerably from an intercept of 0 and a slope of 1.

### 6.3. Selected Error Propagation Illustrations

This section reviews three instances of error propagation. The first, motivated by Koo et al. [46], assesses analytical measurement error, resampling error, and specification error on indices of spatial autocorrelation. The second, inspired by a widespread recognition of the existence of interacting error sources, evaluates a mixture of sampling and specification error latent in georeferenced data. The third illustrates variance inflation, a common geospatial data complication.

#### 6.3.1. Error Propagation to Spatial Autocorrelation Indices

Table 10 summarizes Moran Coefficient (MC) and Geary Ratio (GR) spatial autocorrelation index results by data factor dimensions. With regard to spatial autocorrelation measures, based upon a root mean squared error criterion, the presumably correct statistical distributional assumption results most closely align with the original data results. Sampling error noticeably impacts these measures, with a tendency to decrease them. Specification error markedly impacts these measures, with the beta and uniform distribution assumptions corrupting them far more than the gamma assumption. The MC indicates that the beta, whereas the GR indicates that the uniform, assumption introduces more specification error.

Meanwhile, the standard errors display considerably more of an impact than the spatial autocorrelation indices themselves. Interestingly, the resampling error does not tend to exhibit the closest alignment with the theoretical standard errors reported for the original data. Specification error introduced by the uniform distribution assumption deviates the most, which is rather obvious from a visual inspection of the table entries. The asymptotic standard error (i.e., 0.85) for the MC is consistent with these results; that for the GR (i.e., 0.214) is not.

The general implication here is that error propagation impacts spatial autocorrelation index values and their standard error estimates. Specification error may or may not be a more serious source of this corruption. This topic merits considerably more future research.

#### 6.3.2. Error Propagation from a Mixture of Error Sources

Another experiment, inspired by Gustavsson et al. [47], involved the following two-step procedure: draw a bootstrap census tract tessellation stratified random sample (with replacement) of 3322 log-normal fitted values, and then use these measurements to generate simulated (log-normal distribution assumption) assaying analytical errors. This sequence was executed 10,000 times. Table 11 summarizes output for this combined error source sensitivity analysis. As expected, deviations from the original data results increase, with sampling error markedly dominating analytical measurement error (also see Table 12).

#### 6.3.3. Error and Spatial Autocorrelation Induced Variance Inflation

The final exploratory analysis concerns an additional evaluation of error, partially reminiscent of the arguments of Koo et al. [46]. A principal impact of positive spatial autocorrelation is variance inflation. Therefore, a conventional variance formula includes specification error, whereas adjusting for spatial autocorrelation renders an uninflated variance estimate; adjustment here is with the popular pure spatial simultaneous autoregressive (SAR) model specification. Table 12 shows the relevant results. The various pairs of data results demonstrate how variance inflation increases with increasing positive spatial autocorrelation; this inflation is rather modest for this example because the spatial autocorrelation parameter ρ is not vary large (e.g., approximately 0.4), generating roughly a 20% increase in variance. Random sampling error impacts variance estimates more than the other reported statistical quantities; Table 12 suggests that it also might slightly dampen variance inflation vis-à-vis the other error sources. In contrast, random measurement error has similar but less pronounced impacts on the reported statistical quantities; it appears to have a rather neutral impact on variance inflation. When mixed, random sampling error impacts tend to dominate analytical measurement error impacts. To quantify this situation based on the Table 12 information, for Factor-1.

Total variance − analytical measurement error − sampling error: 1 − 0.002 − 0.122 = 0.876 (with rounding error vis-à-vis Table 12).

The corresponding mixture result confirms this outcome. Results for the other three factors are more ambiguous.

Because the gamma distribution often is an assumption competitor for the log-normal distribution, it furnishes the basis for a minimum specification error assessment here. Specification error impacts are detectable in Factor-1 and considerable in the other three factors. These impacts are at least as substantial as those for sampling error, being far more extreme for Factor-4. Accordingly, in terms of trace metal analytical error, the evidence presented in this paper supports the following rank ordering:

measurement < sampling < specification

This ordering merits further research.

## 7. Discussion

Table 13 furnishes a basic summary revealing prominent similarities and differences displayed by the presence of measurement, sampling, and specification error. The random nature of these error sources partially functions as noise, deteriorating latent patterns. Regardless, Factor-4 is a robust dimension, and Factor-1 is a reasonably robust dimension, with error-created confusion primarily materializing in the second and third factors. Sampling error tends to dominate analytical measurement error, with specification error introducing serious corruption. Meanwhile Rb results appear to be the most sensitive, of the 15 heavy metals studied here, to sources of error.

## 8. Conclusions and Implications

This paper investigates assaying errors in surface soil samples that were collected across the city of Syracuse, NY, mostly during the summers of 2003 and 2004. These samples are composed of a total of 3628 observations, with measurements for 15 heavy metals. The geographic distributions of the 15 heavy metals were examined at the individual point level and at the aggregate census tract levels. In addition, the data analytic dimensions of these heavy metals were examined using varimax-rotated factor analysis. The results show that the assaying errors do not present a high level of spatial autocorrelation. This result may indicate that the assay errors generally are independent across geographic locations. The factor analysis results imply that the geographic distributions of the 15 heavy metals display four prominent data analytic dimensions and that their patterns are associated with objects in the physical environment (e.g., expressways), historical settlement patterns in the city, and low housing/population densities. In addition, these assay error patterns are consistent with those of the corresponding observed values of the 15 heavy metals. Empirically, this outcome shows that the size of assay errors tends to be positively associated with the sizes of observed values.

This paper also investigates impacts of sampling error, measurement error, and their mixture on assaying error for the 15 heavy metals using explanatory simulation experiments. The results show that although both sampling error and measurement error have an impact on assaying errors, measurement error has a greater impact than sampling error. Furthermore, geographically, the measurement error is stable across the census tracts, whereas the sampling error varies considerably. These findings may provide useful insights for constructing a more rigorous sampling design as well as a measurement plan. Also, the results suggest that specification error would have a severe impact; however, this conjecture needs further investigation to better understand this impact in a general context. Finally, the results of the simulation experiments confirm a geographic resolution impact.

Because this paper investigates a specific dataset, the findings may not be directly applicable to other geographic landscapes. Nevertheless, this paper shows impacts of the different error sources on assaying error and posits a potential importance ranking of different error sources.

## Figures and Tables

**Table 1:** Selected relevant Syracuse soil sample attributes for the 15 trace metals.

Trace Metal	Limit of Detection; McComb et al. [35]	US Natural Median Background Level; Smith et al. [36]	World-Wide Average Contamination Level; Kabata-Pendias [37]	Maximum Permissible Level (MPL); Vodyanitskii [27]	The Number of Samples Exceeding the MPL (*n* = 3324)
Arsenic (As)	0.61	5.2	6.83	4.5	3324
Chromium (Cr)	14.06	30	59.5	3.8	3324
Cobalt (Co)	9.01	7.7	11.3	24	3324
Copper (Cu)	6.52	14.4	38.9	3.5	3324
Iron (Fe)	12.4	19,500	22,979 **^‡^**	← δ	0
Lead (Pb)	0.7	18.1	27	55	68
Manganese (Mn)	19.4	492	488	200 (pH < 5.2) **^†^**	8
Mercury (Hg)	2.39	0.02	0.07	1.9	3324
Molybdenum (Mo)	1.2	0.78	1.1	253	0
Nickel (Ni)	14.4	13.5	29	2.6	3324
Rubidium (Rb)	0.35	65.2	68	← δ	0
Selenium (Se)	0.73	0.2	0.44	0.11	3324
Strontium (Sr)	0.33	121	175	← δ	0
Zinc (Zn)	2.41	58	70	16	3324
Zirconium (Zr)	0.96	165 *	267	← δ	0

NOTE: * Makishima et al. [38]; **^†^** https://www.uaex.edu/publications/PDF/FSA-2118.pdf (access on 11 May 2021); **^‡^** imputed value; ← δ this arrow symbol points to the worldwide average contamination level to be used.

**Table 2:** Selected descriptive statistics for the theoretical analytical error of the 15 trace metals measured via Syracuse soil sample assaying (*n* = 3324 soil sample points).

Trace Metal	Mean	Std. Dev.^†^	y_min_	y_max_	Three-Parameter Log-Normal Random Variable					
					$\hat{\delta }$	% Outliers	K-S ^‡^	A-D ^§^	Pseudo-R^2^	
									Transformed	Back-Transformed
Arsenic (As)	15.65	10.02	5.96	155.54	–5.8	6.59	0.040	17.019	0.970	0.907
Chromium (Cr)	106.98	9.13	62.07	174.37	–29.4	5.72	0.054	20.428	0.963	0.955
Cobalt (Co)	140.88	15.83	68.66	274.11	75.6	4.78	0.043	16.180 *	0.969	0.962
Copper (Cu)	55.30	4.94	35.06	101.92	–26.9	5.63	0.075	39.525	0.941	0.922
Iron (Fe)	375.82	42.74	179.71	731.27	283.6	4.84	0.043	16.449 *	0.968	0.962
Lead (Pb)	19.31	12.81	6.93	196.38	–6.8	8.09	0.036	14.383	0.973	0.915
Manganese (Mn)	136.31	13.19	80.00	228.34	–16.7	5.17	0.037	9.527	0.980	0.973
Mercury (Hg)	10.46	0.86	6.75	18.81	–5.0	4.96	0.070	32.511	0.952	0.940
Molybdenum (Mo)	4.29	0.31	2.40	6.14	138.2	5.35	0.059	18.985	0.969	0.969
Nickel (Ni)	72.86	5.64	44.80	112.93	–24.7	5.78	0.072	31.913	0.953	0.949
Rubidium (Rb)	4.77	0.53	2.40	8.28	8.1	6.44	0.025 *	3.318	0.990	0.990
Selenium (Se)	7.52	0.73	4.91	17.79	–4.3	5.81	0.074	40.564	0.926	0.854
Strontium (Sr)	8.32	1.64	4.77	22.81	–4.5	2.14	0.089	51.501	0.941	0.924
Zinc (Zn)	40.90	10.39	25.46	150.04	–25.0	3.88	0.079	47.100	0.937	0.870
Zirconium (Zr)	15.78	1.70	6.66	27.13	0.0	6.14	0.053 *	19.916 *	0.963	0.975

NOTE: **^†^** Appendix A displays sample analytical measurement error boxplots; **^‡^** K-S denotes Kolmogorov-Smirnov; ^§^ A-D denotes Anderson-Darling; * Overall, a fitted log-normal corresponded more closely than a fitted gamma distribution (an asterisk denotes the exceptions to this finding); outlier identification resulted from robust estimation using an M-estimator coupled with Huber’s weight and scale.

**Table 3:** Spatial autocorrelation index values for log-measurement errors, $\mathrm{LN}\left(y+\hat{\delta }\right)$.

Trace Metal	Individual Point Data: Ordinary Kriging and K Bessel Function		Census Tract Aggregated Data	
	Effective Range (Meters; Maximum = 14,800)	Cross-Validation Standardized RMSE	MC (MC_max_ = 1.018)	GR (GR_min_ = 0.051)
Arsenic (As)	7035	0.92	0.216	0.691
Chromium (Cr)	7066	1.011	0.11	1.076
Cobalt (Co)	7880	1.048	0.22	0.922
Copper (Cu)	6382	1.004	0.219	0.967
Iron (Fe)	7794	1.051	0.219	0.913
Lead (Pb)	38	0.893	0.215	0.692
Manganese (Mn)	28	0.938	0.086	1.067
Mercury (Hg)	5140	1.005	0.183	0.977
Molybdenum (Mo)	1713	1.033	0.218	0.752
Nickel (Ni)	6977	1.029	0.195	0.963
Rubidium (Rb)	809	1.015	0.423	0.607
Selenium (Se)	33	0.869	0.162	0.91
Strontium (Sr)	6465	1.116	0.207	0.851
Zinc (Zn)	38	0.818	0.244	0.745
Zirconium (Zr)	1430	1.056	0.349	0.558

NOTE: log-transformed values more closely conform to a bell-shaped curve but fail to markedly change the spatial autocorrelation index values; MC denotes Moran’s *I* (i.e., the Moran coefficient), and GR denotes Geary’s *c* (i.e., the Geary Ratio), which are the two most popular spatial autocorrelation indices.

**Table 4:** Syracuse trace metal analytical error varimax-rotated factor dimensions.

Trace Metal	Individual Point Data				Census Tract Aggregated Data			
	Factor-1	Factor-2	Factor-3	Factor-4	Factor-1	Factor-2	Factor-3	Factor-4
Arsenic (As)	0.181	0.193	0.95	0.067	0.193	0.203	0.957	0.026
Chromium (Cr)	0.721	0.542	0.299	0.112	0.893	0.367	0.207	0.077
Cobalt (Co)	0.313	0.901	0.171	0.109	0.416	0.835	0.273	0.071
Copper (Cu)	0.859	0.313	0.266	0.035	0.96	0.157	0.178	−0.027
Iron (Fe)	0.3	0.909	0.149	0.105	0.392	0.847	0.266	0.058
Lead (Pb)	0.179	0.19	0.951	0.069	0.188	0.199	0.959	0.028
Manganese (Mn)	0.346	0.801	0.232	0.15	0.52	0.694	0.304	0.125
Mercury (Hg)	0.822	0.329	0.379	0.098	0.944	0.192	0.205	−0.010
Molybdenum (Mo)	0.137	0.259	0.079	0.938	0.191	0.119	0.085	0.958
Nickel (Ni)	0.828	0.416	0.264	0.085	0.942	0.244	0.165	−0.013
Rubidium (Rb)	−0.091	0.827	0.209	0.183	−0.419	0.779	0.042	−0.073
Selenium (Se)	0.674	0.307	0.602	0.114	0.852	0.202	0.441	0.009
Strontium (Sr)	0.875	−0.144	0.043	−0.110	0.892	−0.286	0.1	−0.073
Zinc (Zn)	0.386	0.211	0.811	−0.015	0.64	0.212	0.702	−0.053
Zirconium (Zr)	−0.069	0.096	0.03	0.977	−0.242	−0.047	−0.056	0.952
% variance	29.3	26.3	22.4	13.2	42.4	20.2	19.5	12.5

**Table 5:** Log-normal distribution simulated Syracuse trace metal analytical assay measurement error varimax-rotated factor dimensions: census tract averaged soil sample data (10,000 replications).

Trace Metal	Factor-1		Factor-2		Factor-3		Factor-4	
	$\bar{r}$	$s_{r}$	$\bar{r}$	$s_{r}$	$\bar{r}$	$s_{r}$	$\bar{r}$	$s_{r}$
Arsenic (As)	0.192	0.01	0.291	0.243	**0.866**	0.243	0.026	0.01
Chromium (Cr)	**0.89**	0.008	0.35	0.053	0.222	0.048	0.077	0.013
Cobalt (Co)	0.414	0.017	**0.77**	0.178	0.334	0.177	0.071	0.014
Copper (Cu)	**0.956**	0.005	0.16	0.017	0.173	0.017	−0.027	0.014
Iron (Fe)	0.39	0.017	**0.78**	0.184	0.33	0.184	0.057	0.015
Lead (Pb)	0.187	0.01	0.288	0.244	**0.867**	0.245	0.028	0.009
Manganese (Mn)	0.518	0.017	**0.65**	0.123	0.345	0.121	0.124	0.015
Mercury (Hg)	**0.94**	0.005	0.194	0.016	0.201	0.016	−0.01	0.014
Molybdenum (Mo)	0.19	0.015	0.115	0.017	0.088	0.016	**0.955**	0.004
Nickel (Ni)	**0.938**	0.005	0.236	0.03	0.172	0.025	−0.013	0.014
Rubidium (Rb)	−0.418	0.016	**0.689**	0.239	0.13	0.24	−0.073	0.011
Selenium (Se)	**0.846**	0.013	0.23	0.081	0.409	0.082	0.009	0.018
Strontium (Sr)	**0.888**	0.011	−0.238	0.126	0.052	0.13	−0.072	0.016
Zinc (Zn)	0.635	0.018	0.271	0.163	**0.639**	0.162	−0.053	0.017
Zirconium (Zr)	−0.24	0.014	−0.049	0.014	−0.054	0.014	**0.949**	0.004
% variance	42.1		20.1		19.4		12.4	

NOTE: bold font denotes a prominent factor loading (see Table 4); $s_{r}$ denotes the standard deviation of a simulated assaying data correlation coefficient.

**Table 6:** Resampled Syracuse trace metal analytical measurement error varimax-rotated factor dimensions: census tract averaged soil sample data (10,000 replications).

Trace Metal	Factor-1		Factor-2		Factor-3		Factor-4	
	$\bar{r}$	$s_{r}$	$\bar{r}$	$s_{r}$	$\bar{r}$	$s_{r}$	$\bar{r}$	$s_{r}$
Arsenic (As)	0.206	0.036	0.522	0.371	**0.625**	0.381	0.026	0.037
Chromium (Cr)	**0.887**	0.03	0.322	0.089	0.248	0.072	0.071	0.036
Cobalt (Co)	0.436	0.108	**0.612**	0.286	0.458	0.264	0.075	0.064
Copper (Cu)	**0.953**	0.026	0.182	0.056	0.149	0.071	−0.036	0.043
Iron (Fe)	0.414	0.113	**0.615**	0.296	0.457	0.275	0.062	0.068
Lead (Pb)	0.201	0.036	0.521	0.374	**0.625**	0.384	0.029	0.036
Manganese (Mn)	0.521	0.086	**0.55**	0.204	0.415	0.183	0.123	0.069
Mercury (Hg)	**0.934**	0.026	0.217	0.053	0.186	0.07	−0.008	0.041
Molybdenum (Mo)	0.181	0.062	0.119	0.059	0.091	0.096	**0.944**	0.09
Nickel (Ni)	**0.934**	0.025	0.229	0.061	0.18	0.058	−0.008	0.036
Rubidium (Rb)	−0.346	0.108	0.461	0.358	0.376	0.383	−0.013	0.105
Selenium (Se)	**0.838**	0.033	0.318	0.133	0.324	0.159	0.018	0.034
Strontium (Sr)	**0.867**	0.064	−0.117	0.198	−0.073	0.219	−0.096	0.069
Zinc (Zn)	**0.618**	0.048	0.429	0.253	0.473	0.277	−0.058	0.043
Zirconium (Zr)	−0.224	0.049	−0.040	0.042	−0.030	0.101	**0.944**	0.087
% variance	41.7		20.8		18.8		12.6	

NOTE: bold font denotes a prominent factor loading (see Table 4); S_r_ denotes the standard deviation of a resample correlation coefficient.

**Table 7:** Syracuse trace metal induced gamma distributed measurement error varimax-rotated factor dimensions: census tract averaged soil sample data (10,000 replications).

Trace Metal	Factor-1		Factor-2		Factor-3		Factor-4	
	$\bar{r}$	$s_{r}$	$\bar{r}$	$s_{r}$	$\bar{r}$	$s_{r}$	$\bar{r}$	$s_{r}$
Arsenic (As)	0.079	0.095	0.38	0.319	**0.505**	0.366	0.108	0.197
Chromium (Cr)	**0.846**	0.066	0.273	0.151	0.128	0.098	0.069	0.08
Cobalt (Co)	0.5	0.171	**0.569**	0.282	0.24	0.198	0.104	0.148
Copper (Cu)	**0.884**	0.072	0.162	0.163	0.056	0.114	0	0.087
Iron (Fe)	0.483	0.175	**0.576**	0.289	0.233	0.209	0.089	0.154
Lead (Pb)	0.271	0.075	0.396	0.292	0.478	0.396	0.033	0.231
Manganese (Mn)	**0.518**	0.15	0.499	0.207	0.257	0.145	0.162	0.131
Mercury (Hg)	**0.859**	0.07	0.187	0.152	0.1	0.133	0.005	0.097
Molybdenum (Mo)	0.177	0.081	0.105	0.137	0.215	0.351	**0.686**	0.36
Nickel (Ni)	**0.867**	0.066	0.196	0.157	0.064	0.107	−0.012	0.084
Rubidium (Rb)	−0.270	0.186	0.418	0.416	0.151	0.351	−0.047	0.294
Selenium (Se)	**0.77**	0.081	0.256	0.154	0.218	0.209	0.017	0.132
Strontium (Sr)	**0.685**	0.161	−0.154	0.25	−0.173	0.168	−0.180	0.13
Zinc (Zn)	0.043	0.043	0.146	0.245	0.296	0.296	0.084	0.294
Zirconium (Zr)	−0.229	0.075	−0.080	0.139	0.13	0.378	**0.654**	0.37
% variance	34.9		16.9		12.9		11.1	

NOTE: bold font denotes a prominent factor loading (see Table 4); S_r_ denotes the standard deviation of a combined simulated assaying and resampling error correlation coefficient.

**Table 8:** Syracuse trace metal induced beta distributed measurement error varimax-rotated factor dimensions: census tract averaged soil sample data (10,000 replications).

Trace Metal	Factor-1		Factor-2		Factor-3		Factor-4	
	$\bar{r}$	$s_{r}$	$\bar{r}$	$s_{r}$	$\bar{r}$	$s_{r}$	$\bar{r}$	$s_{r}$
Arsenic (As)	0.211	0.149	**0.636**	0.355	0.353	0.381	0.007	0.125
Chromium (Cr)	**0.75**	0.117	0.33	0.161	0.212	0.176	0.014	0.18
Cobalt (Co)	**0.498**	0.242	0.405	0.257	0.373	0.304	0.04	0.188
Copper (Cu)	**0.752**	0.149	0.325	0.168	0.161	0.241	−0.046	0.209
Iron (Fe)	0.474	0.253	0.388	0.267	0.366	0.312	0.029	0.199
Lead (Pb)	0.206	0.151	**0.634**	0.359	0.352	0.383	0.009	0.125
Manganese (Mn)	**0.557**	0.202	0.372	0.225	0.303	0.234	0.058	0.188
Mercury (Hg)	0.489	0.238	0.17	0.228	0.114	0.234	0.03	0.364
Molybdenum (Mo)	0.031	0.189	0.021	0.214	0.016	0.271	**0.494**	0.457
Nickel (Ni)	**0.755**	0.137	0.306	0.17	0.174	0.205	−0.028	0.203
Rubidium (Rb)	−0.099	0.305	0.09	0.374	0.292	0.55	0.025	0.292
Selenium (Se)	0.433	0.225	0.258	0.221	0.141	0.238	0.038	0.368
Strontium (Sr)	**0.601**	0.273	0.223	0.278	−0.009	0.44	−0.039	0.235
Zinc (Zn)	0.492	0.117	**0.57**	0.221	0.29	0.305	−0.046	0.187
Zirconium (Zr)	−0.164	0.168	−0.092	0.173	−0.045	0.217	0.359	0.485
% variance	28.3		20.2		16.1		10.2	

NOTE: bold font denotes a prominent factor loading (see Table 4); S_r_ denotes the standard deviation of a combined simulated assaying and resampling error correlation coefficient.

**Table 9:** Syracuse trace metal induced uniform distributed measurement error varimax-rotated factor dimensions: census tract averaged soil sample data (10,000 replications).

Trace Metal	Factor-1		Factor-2		Factor-3		Factor-4	
	$\bar{r}$	$s_{r}$	$\bar{r}$	$s_{r}$	$\bar{r}$	$s_{r}$	$\bar{r}$	$s_{r}$
Arsenic (As)	0.274	6.0 × 10^−5^	**0.949**	2.0 × 10^−5^	0.135	7.0 × 10^−5^	−0.032	7.0 × 10^−5^
Chromium (Cr)	**0.888**	8.0 × 10^−5^	0.197	1.4 × 10^−4^	0.368	1.8 × 10^−4^	0.006	1.5 × 10^−4^
Cobalt (Co)	0.433	1.5 × 10^−4^	0.302	1.1 × 10^−4^	**0.82**	1.0 × 10^−4^	0.049	1.3 × 10^−4^
Copper (Cu)	**0.924**	5.0 × 10^−5^	0.283	1.2 × 10^−4^	0.149	1.4 × 10^−4^	−0.148	1.3 × 10^−4^
Iron (Fe)	0.412	1.5 × 10^−4^	0.293	1.2 × 10^−4^	**0.83**	9.0 × 10^−5^	0.035	1.3 × 10^−4^
Lead (Pb)	0.271	6.0 × 10^−5^	**0.95**	2.0 × 10^−5^	0.13	7.0 × 10^−5^	−0.026	7.0 × 10^−5^
Manganese (Mn)	0.614	1.9 × 10^−4^	0.185	1.7 × 10^−4^	**0.65**	1.8 × 10^−4^	0.124	1.9 × 10^−4^
Mercury (Hg)	**0.927**	5.0 × 10^−5^	0.259	1.3 × 10^−4^	0.144	1.4 × 10^−4^	−0.119	1.4 × 10^−4^
Molybdenum (Mo)	0.066	1.2 × 10^−4^	−0.01	1.1 × 10^−4^	0.051	1.2 × 10^−4^	**0.978**	3.0 × 10^−5^
Nickel (Ni)	**0.926**	6.0 × 10^−5^	0.19	1.4 × 10^−4^	0.252	1.6 × 10^−4^	−0.106	1.5 × 10^−4^
Rubidium (Rb)	−0.268	1.3 × 10^−4^	−0.105	1.0 × 10^−4^	**0.863**	7.0 × 10^−5^	−0.067	1.2 × 10^−4^
Selenium (Se)	**0.87**	7.0 × 10^−5^	0.422	1.2 × 10^−4^	0.138	1.3 × 10^−4^	−0.102	1.2 × 10^−4^
Strontium (Sr)	**0.874**	7.0 × 10^−5^	0.272	1.1 × 10^−4^	−0.304	1.7 × 10^−4^	−0.029	1.2 × 10^−4^
Zinc (Zn)	0.611	1.0 × 10^−4^	**0.744**	8.0 × 10^−5^	0.116	1.1 × 10^−4^	−0.166	1.0 × 10^−4^
Zirconium (Zr)	−0.335	1.1 × 10^−4^	−0.11	1.0 × 10^−4^	−0.023	1.0 × 10^−4^	**0.916**	5.0 × 10^−5^
% variance	42.2		20.4		19.6		12.7	

NOTE: bold font denotes a prominent factor loading (see Table 4); S_r_ denotes the standard deviation of a combined simulated assaying and resampling error correlation coefficient.

**Table 10:** Factor score spatial autocorrelation index values for the various datasets (Table 5, Table 6, Table 7, Table 8 and Table 9).

Error Source	Factor-1		Factor-2		Factor-3		Factor-4	
	MC	GR	MC	GR	MC	GR	MC	GR
Original data	0.184	1.039	0.242	0.8	0.162	0.724	0.3	0.617
	−0.078	−0.129	−0.081	−0.098	−0.081	−0.097	−0.081	−0.098
Analytical (log-normal assumption)	0.184	1.038	0.231	0.793	0.172	0.733	0.297	0.62
	−0.01	−0.013	−0.026	−0.028	−0.028	−0.024	−0.011	−0.012
Resampling	0.153	1.048	0.176	0.807	0.18	0.78	0.235	0.696
	−0.06	−0.086	−0.058	−0.072	−0.07	−0.064	−0.059	−0.064
Gamma assumption	0.195	1.04	0.205	0.808	0.165	0.779	0.202	0.766
	−0.05	−0.074	−0.074	−0.073	−0.078	−0.063	−0.077	−0.064
Beta assumption	0.132	0.989	0.189	0.74	0.218	0.746	0.088	0.882
	−0.085	−0.122	−0.062	−0.131	−0.089	−0.107	−0.12	−0.135
Uniform assumption	0.178	0.899	0.265	0.491	0.317	0.718	0.289	0.648
	(<0.001)	(<0.001)	(<0.001)	(<0.001)	(<0.001)	(<0.001)	(<0.001)	(<0.001)
Mixture	0.152	1.048	0.174	0.806	0.182	0.78	0.235	0.698
	−0.062	−0.088	−0.058	−0.074	−0.062	−0.064	−0.059	−0.063

NOTE: standard errors appear in parentheses; the original data standard errors assume randomization (normality respectively renders standard errors of 0.081 and 0.099).

**Table 11:** Combined log-normal simulation and resampled Syracuse trace metal analytical error varimax-rotated factor dimensions: census tract averaged soil sample data (10,000 replications).

Trace Metal	Factor-1		Factor-2		Factor-3		Factor-4	
	$\bar{r}$	$s_{r}$	$\bar{r}$	$s_{r}$	$\bar{r}$	$s_{r}$	$\bar{r}$	$s_{r}$
Arsenic (As)	0.205	0.038	0.539	0.373	**0.606**	0.384	0.026	0.038
Chromium (Cr)	**0.884**	0.033	0.319	0.093	0.248	0.077	0.071	0.038
Cobalt (Co)	0.439	0.115	**0.596**	0.289	0.466	0.267	0.076	0.068
Copper (Cu)	**0.948**	0.03	0.185	0.062	0.145	0.077	−0.036	0.045
Iron (Fe)	0.417	0.12	**0.598**	0.299	0.465	0.279	0.062	0.073
Lead (Pb)	0.201	0.038	0.537	0.376	**0.605**	0.387	0.029	0.037
Manganese (Mn)	0.523	0.091	0.539	0.207	0.419	0.186	0.122	0.072
Mercury (Hg)	**0.93**	0.03	0.22	0.059	0.183	0.077	−0.007	0.043
Molybdenum (Mo)	0.181	0.063	0.117	0.059	0.093	0.103	**0.941**	0.097
Nickel (Ni)	**0.93**	0.029	0.229	0.066	0.179	0.065	−0.009	0.038

NOTE: bold font denotes a prominent factor loading (see Table 4); S_r_ denotes the standard deviation of a combined simulated assaying and resampling error correlation coefficient.

**Table 12:** Factor variance estimates based upon a spatial simultaneous autoregressive model specification and census tract aggregated heavy metal results.

Dataset	Parameter	Factor-1	Factor-2	Factor-3	Factor-4
Original (Table 4)	${\hat{\sigma }}^{2}$	1	1	1	1
	$\hat{\rho }$	0.264	0.435	0.527	0.484
	${\hat{\sigma }}_{\mathrm{adjusted}}^{2}$	0.928	0.832	0.787	0.785
	Pr(S-W)	<0.0001	0.575	0.961	0.444
Analytical measurement error (Table 5)	${\hat{\sigma }}^{2}$	0.998	0.786	0.783	0.993
	$\hat{\rho }$	0.264	0.442	0.532	0.484
	${\hat{\sigma }}_{\mathrm{adjusted}}^{2}$	0.926	0.649	0.611	0.781
	Pr(S-W)	<0.0001	0.607	0.812	0.443
Sampling error (Table 6)	${\hat{\sigma }}^{2}$	0.878	0.439	0.415	0.777
	$\hat{\rho }$	0.247	0.482	0.532	0.484
	${\hat{\sigma }}_{\mathrm{adjusted}}^{2}$	0.821	0.351	0.319	0.611
	Pr(S-W)	<0.0001	0.893	0.604	0.455
Mixture (Table 11)	${\hat{\sigma }}^{2}$	0.875	0.435	0.413	0.774
	$\hat{\rho }$	0.246	0.486	0.529	0.487
	${\hat{\sigma }}_{\mathrm{adjusted}}^{2}$	0.818	0.356	0.319	0.606
	Pr(S-W)	<0.0001	0.896	0.594	0.451
Specification (Table 7)	${\hat{\sigma }}^{2}$	0.889	0.456	0.343	0.488
	$\hat{\rho }$	0.282	0.460	0.426	0.435
	${\hat{\sigma }}_{\mathrm{adjusted}}^{2}$	0.816	0.365	0.291	0.403
	Pr(S-W)	<0.0001	0.856	0.133	0.985

**Table 13:** Syracuse trace metal analytical error varimax-rotated factor dimension comparisons (Table 4, Table 5, Table 6, Table 7, Table 8 and Table 9 and Table 11).

Trace Metal	Original Data		Census Tract Averaged Simulated Data					
	Points	Census Tracts	Sampling Error	Measurement Error	Specification Error			Mixture
					Gamma	Beta	Uniform	
Arsenic (As)	3	3	3	3	3	2	2	3
Chromium (Cr)	1	1	1	1	1	1	1	1
Cobalt (Co)	2	2	2	2	2	1	3	2
Copper (Cu)	1	1	1	1	1	1	1	1
Iron (Fe)	2	2	2	2	2	—	3	2
Lead (Pb)	3	3	3	3	—	2	2	3
Manganese (Mn)	2	2	2	2	1	1	3	—
Mercury (Hg)	1	1	1	1	1	—	1	1
Molybdenum (Mo)	4	4	4	4	4	4	4	4
Nickel (Ni)	1	1	1	1	1	1	1	1
Rubidium (Rb)	2	2	2	—	—	—	3	—
Selenium (Se)	1	1	1	1	1	—	1	1
Strontium (Sr)	1	1	1	1	1	1	1	1
Zinc (Zn)	3	3	3	1	—	2	2	1
Zirconium (Zr)	4	4	4	4	4	—	4	4
Threshold $\left|\bar{r}\right|$	0.674	0.694	0.639	0.612	0.505	0.494	0.744	0.596
% variance	91.2	93.6	94	93.9	75.8	74.8	94.9	93.3

NOTE: — denotes no factor loading (i.e., correlation) greater than the stipulated threshold value.

**Figure A1:**
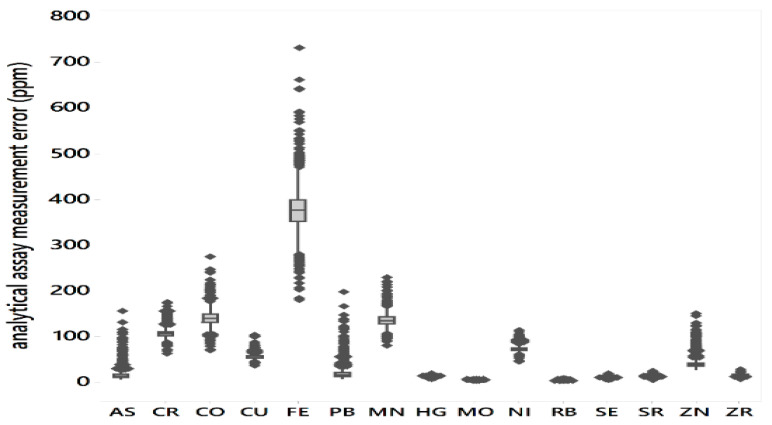
Sample analytical assay measurement error boxplots.

## Data Availability

Restrictions apply to the availability of the soil sample data. These data, now housed at UTD, are available by request in census tract aggregated form, as per presiding agreements established by IRBs in coordination with the Onondaga County Health Department.

## Appendix B. Tabulation of Important Back-Transformation Statistics

Griffith [48] discusses back-transformations, specifically commenting about transformation-introduced specification error that can be indexed by the mean of the ratio ${\hat{y}}_{i}/y_{I}$ appearing in Table A1. All of the Syracuse heavy metals contain less than 1% specification error based upon this criterion. Furthermore, 11 of the heavy metals have a bivariate regression slope of approximately 1, its expectation for a perfect match between the two sets of values. In particular, Zn exhibits what appears to be excessive bivariate regression parameter values, implying that its log-normal characterization may contain excessive specification error.

**Table A1:** Selected descriptive statistics for calculating and assessing the theoretical error of the 15 trace metals measured via Syracuse soil sample assaying.

Trace Metal	$LN\left(y_{i}+\delta \right)=\alpha +\beta \frac{\left(r_{i}-3/8\right)}{\left(n+1/4\right)}+\varepsilon_{i}$ , i=1,2,⋯,n		MSE	${\hat{y}}_{i}/y_{i}$	
	$\hat{\alpha }$	$\hat{\beta }$		Mean	Standard Deviation
Arsenic (As)	−5.817	1.406	0.01274	1.006	0.065
Chromium (Cr)	−2.294	1.022	0.00048	1.000	0.016
Cobalt (Co)	−0.176	1.001	0.00017	1.000	0.020
Copper (Cu)	−3.321	1.060	0.00153	1.000	0.020
Iron (Fe)	−0.147	1.000	0.00013	1.000	0.021
Lead (Pb)	−6.794	1.386	0.01214	1.006	0.063
Manganese (Mn)	−0.936	1.007	0.00023	1.000	0.013
Mercury (Hg)	−0.464	1.045	0.00109	1.000	0.017
Molybdenum (Mo)	0.001	1.000	0.00000	1.000	0.016
Nickel (Ni)	−2.028	1.028	0.00060	1.000	0.016
Rubidium (Rb)	−0.002	1.000	0.00002	1.000	0.012
Selenium (Se)	−0.742	1.099	0.00285	1.001	0.024
Strontium (Sr)	−1.547	1.189	0.00722	1.002	0.039
Zinc (Zn)	−13.797	1.347	0.01302	1.002	0.052
Zirconium (Zr)	0.502	0.968	0.00046	1.000	0.024

## Appendix C. A Cross-Tabulation of Heavy Metals and Their Potential Sources

This cross-tabulation reflects findings by Wuana et al. [49], Aschale et al. [50], and Smiljanić et al. [51].

**Table A2:** Cross-tabulation of selected trace metals and their potential yard landscape sources.

Heavy Metal	Fertilizers	Pesticides	Preservatives	Biosolids/Manures	Storm Water	Vehicles
Arsenic (As)	X	X	X	X	X	
Chromium (Cr)		X	X	X		X
Cobalt (Co)	X			X		
Copper (Cu)	X	X	X			X
Iron (Fe)						X
Lead (Pb)	X	X	X	X	X	X
Manganese (Mn)	X	X				
Mercury (Hg)	X	X		X	X	
Molybdenum (Mo)				X		X
Nickel (Ni)	X			X		X
Rubidium (Rb)						
Selenium (Se)	X			X		
Strontium (Sr)						
Zinc (Zn)	X	X		X		X
Zirconium (Zr)						X

## Appendix E. Random Variable Distributions Selected to Explore Specification Error

**Table A3:** Selected descriptive statistics for the theoretical analytical error of the 15 trace metals measured via Syracuse soil sample assaying (*n* = 3324 soil sample points).

Trace Metal	Gamma Distribution					Beta Distribution (ppm)				Uniform Distribution		
	$\hat{\alpha }$ (Shape)		$\hat{\beta }$ (Scale)	$\hat{\delta }$	Pseudo-R^2^	$\hat{\alpha }$		$\hat{\beta }$	Pseudo-R^2^	y_min_	y_max_	R^2^
	min	max				min	max					
Arsenic (As)	0.8	68.0	4.752	−5	0.93	0.4	35.2	282,883	0.66	5	156	0.45
Chromium (Cr)	37.8	106.8	1.317	−46	0.95	85.0	187.4	1,194,207	0.98	62	175	0.82
Cobalt (Co)	127.3	230.1	1.318	47	0.91	39.9	114.1	492,617	0.99	68	275	0.85
Copper (Cu)	20.1	77.6	0.927	−31	0.97	88.1	195.9	2,394,069	0.93	35	102	0.76
Iron (Fe)	171.9	287.2	3.091	210	0.91	26.2	76.2	129,665	0.99	179	732	0.85
Lead (Pb)	0.7	73.6	6.155	−6	0.91	0.3	34.3	212,688	0.66	6	197	0.45
Manganese (Mn)	44.0	116.5	1.792	−41	0.93	58.6	144.9	689,516	0.99	80	229	0.86
Mercury (Hg)	22.2	82.4	0.155	−6	0.97	9.7	20.7	1,361,555	0.95	6	19	0.78
Molybdenum (Mo)	393.2	555.6	0.015	2	0.85	0.8	1.5	248,568	0.98	2	7	0.87
Nickel (Ni)	38.0	106.4	0.815	−35	0.96	113.4	231.4	2,239,631	0.97	44	113	0.81
Rubidium (Rb)	336.2	495.1	0.027	5	0.92	48.5	137.6	17,260,264	1.00	2	9	0.91
Selenium (Se)	18.0	66.1	0.151	−4	0.95	8.1	18.5	1,635,145	0.86	4	18	0.66
Strontium (Sr)	5.0	49.3	0.533	−4	0.99	14.0	73.2	3,905,656	0.84	4	23	0.66
Zinc (Zn)	2.0	56.6	3.846	−25	0.96	8.6	58.6	568,891	0.73	25	151	0.52
Zirconium (Zr)	340.0	490.1	0.090	17	0.88	51.2	141.4	5,433,130	0.99	6	28	0.91

**Table A4:** Bivariate regression coefficients for response variable y and covariate $\hat{y}$ (predicted with the quantile covariate) from Table A3.

Trace Metal	Gamma Distribution		Beta Distribution (ppm)		Uniform Distribution (0, 1)	
	a	b	a × 10^−8^	b	a	b
Arsenic (As)	3.08114	0.79557	−10.7	1504.28	3.08813	0.15557
Chromium (Cr)	3.53192	0.96686	−3.0	1668.71	76.77784	0.25547
Cobalt (Co)	3.59791	0.97439	−9.7	2909.04	98.63300	0.24652
Copper (Cu)	0.77400	0.98592	−1.7	881.54	40.06520	0.22243
Iron (Fe)	8.33863	0.97776	−26.7	7855.31	262.77582	0.24818
Lead (Pb)	4.72482	0.74484	−13.9	1930.21	3.24790	0.15801
Manganese (Mn)	6.74299	0.95033	−6.3	2429.51	92.13916	0.28651
Mercury (Hg)	0.29654	0.97152	−0.2	155.93	7.65060	0.21986
Molybdenum (Mo)	0.10814	0.97480	0.0	57.40	3.14011	0.27041
Nickel (Ni)	1.92246	0.97352	−1.1	1026.11	52.52146	0.25786
Rubidium (Rb)	0.11093	0.97669	−0.3	97.37	3.18473	0.29627
Selenium (Se)	−0.29095	1.03881	−0.3	124.85	5.72063	0.15852
Strontium (Sr)	0.16369	0.98023	−1.4	277.49	4.79623	0.25584
Zinc (Zn)	1.90502	0.95328	−9.3	1637.74	22.63288	0.20818
Zirconium (Zr)	0.45664	0.97102	−1.0	312.82	11.15278	0.27411

NOTE: because its results are based on ppm, the beta distribution regression slope should be compared with 1000, whereas the other two slopes should be compared with 1; a and b in the column headings denote parameter coefficients for the intercept and covariate in the bivariate regression.
